# Compact and low cross-polarization microstrip patch antenna for 5G Internet of Things applications

**DOI:** 10.1371/journal.pone.0341549

**Published:** 2026-02-11

**Authors:** Hoang Nguyen-Huy, Hung Tran, Hung Pham-Duy, Tan Dao-Duc, Niamat Hussain

**Affiliations:** 1 Faculty of Electrical and Electronic Engineering, PHENIKAA School of Engineering, PHENIKAA University, Yen Nghia, Ha Dong, Hanoi, Vietnam; 2 Department of Convergence Engineering for Intelligent Drone, Sejong University, Seoul, Republic of Korea; 3 James Watt School of Engineering, University of Glasgow, Scotland, United Kingdom; Parul University Parul Institute of Technology, INDIA

## Abstract

This paper presents a miniaturization technique for microstrip patch antennas operating at the fundamental *TM*_01_ mode based on a loading capacitor approach. Several gaps are inserted into the patch, and with the aid of shorting vias, coupled gaps are produced. These function as a slow-wave transmission line, and then, the operating frequency is significantly moved to a lower frequency range. The final radiating element with dimensions of approximately quarter-effective wavelength at 4.5 GHz exhibits good matching performance in the frequency range from 4.48 to 4.52 GHz and broadside gain around 5.0 dBi. It is noted that the proposed design exhibits extremely low cross-polarization radiation of less than –40 dB, which is much lower than that of the conventional quarter-wavelength patch antenna. In comparison with the reported compact patch antennas in literature, the proposed miniaturized patch is one of the smallest design with the smallest cross-polarization level, while having comparable gain. Such operation characteristics demonstrate the suitability of the proposed antenna for compact Internet of Things (IoTs) applications working at 5G New Radio *n*79 band.

## Introduction

With the rapid development of the Internet of Things (IoTs), there is a strong demand for antenna miniaturization as wireless gadgets are getting smaller [1,2]. Among various types of antennas, microstrip patches have been widely adopted in communication systems due to their advantages of lightweight, low profile, as well as ease of integration [3]. The conventional microstrip patch works at the fundamental mode of *TM*_01_ with an electrical length of about half a wavelength. The most common method for achieving patch size reduction is to use a high permittivity substrate [4–6], but the miniaturization degree is limited. Alternatively, a higher miniaturization degree can be obtained by using defected ground structure (DGS) and artificial ground plane [7,8]. In [9,10], irregular ground planes with strip loading can also be a possible technique for patch miniaturization. Alternatively, using high-profile with multiple shorting vias is also an effective method for size reduction [11,12]. The critical drawbacks of such methods are high cost and/or complicated structure, which makes them not suitable for consumer applications. The most effective method is to modify the radiating element, rather than modifying the ground plane. A possible method is to replace the straight resonant edge of the patch with a meandered [13–15], curved edges [16,17], and fractal patch [18,19] for size reduction of about 40%. Larger size reduction can be achieved in [20–24]. Here, different slow-wave structures, including periodic and non-periodic structures, are embedded into the patches. Cutting the radiating patch with various shaped slots is another effective method [25,26]. Overall, although these techniques are helpful for miniaturization, they have drawbacks such as a limited miniaturization degree and/or high cross-polarization radiation. Besides, using differential feeding techniques or complicated ground structures is also another disadvantage of specific designs.

A quarter-wavelength patch antenna is a promising candidate for compact design. By using the shorting pins or plate [27,28], the patch size can be reduced from half-wavelength to quarter-wavelength, while achieving the same radiation feature. Nonetheless, this method causes a large cross-polarization level due to the unequal equivalent magnetic currents on the non-radiating edges. Several methods have been published to suppress the cross-polarization radiation [29–31]. To conclude, despite compact size being obtained, complicated structure and/or high cross polarization are the current drawbacks of these designs.

This paper presents a low cross-polarization miniaturized patch antenna with an electrical length of about a quarter wavelength. The miniaturization is accomplished by loading several coupled gaps on the patch, which are realized with the aid of shorting vias. The final design with overall dimensions of 0.45λ × 0.45λ × 0.02λ and patch dimensions of 0.15λ × 0.15λ at 4.5 GHz achieves a broadside gain of 5.3 dBi and extremely low cross-polarization radiation of less than –45 dB. Further investigation also indicates that by loading more capacitor, lower operating frequency can be observed, while achieving extremely low cross-polarization level. It is worth noting that despite using the similar capacitive loading method as [20–24], the contribution of the proposed antenna comes from a number of gaps and gap positions, which are different from the others. Thus, the proposed antenna can achieve either smaller size or smaller cross polarization. Compared with the quarter-effective wavelength patch [29–31], these patches inherently possess compact size features. However, the asymmetrical geometry results in very high cross polarization level. This is the critical drawback of such designs in comparison with the proposed one.

## Antenna design and performance

**Fig 1:**
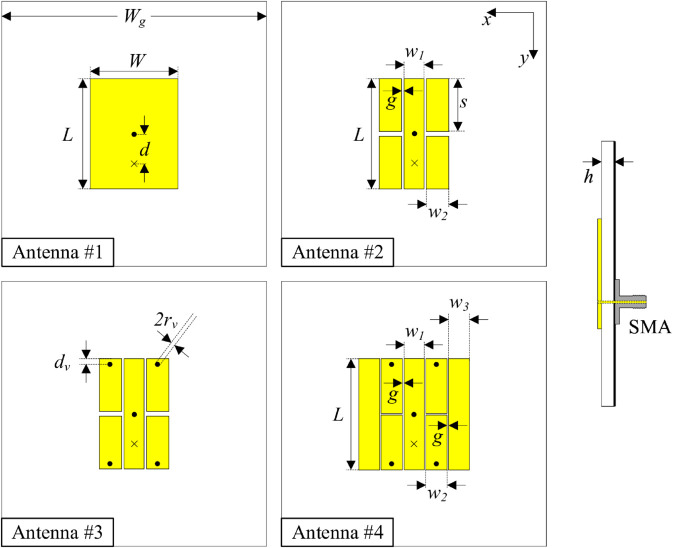
Antenna design evolution.

Fig 1 shows the configurations of four different antennas printed on the top side of a Taconic RF-35 substrate with a dielectric constant of 3.5. Noted that the resonant lengths of these antennas are fixed at 9.8 mm. Antenna #1 is the conventional patch antenna, and its fundamental operating mode is *TM*_01_. Then, several gaps are introduced to the patch, designated as Antenna #2. Antenna #3 is realized with the presence of two shorting pins. The final realization of miniaturized Antenna #4 is achieved by adding more gaps. The optimized design parameters of four antennas are given in Table 1. The ground plane size is chosen at 30 mm × 30 mm. Larger values do not bring further effect on the antenna performance.

**Table 1:** Optimal geometry of different antennas.

Para. (mm)	Antenna #1	Antenna #2	Antenna #3	Antenna #4
*L*	9.8	9.8	9.8	9.8
*W*	6.8			
*d*	2	0.8	1.0	1.8
*s*		4.8	4.8	4.8
*g*		0.14	0.14	0.14
*w*_1_		1.8	1.8	1.8
*w*_2_		2.0	2.0	1.86
$d_{v}$			0.5	0.5
$r_{v}$			0.2	0.2
*w*_3_				1.86

**Fig 2:**
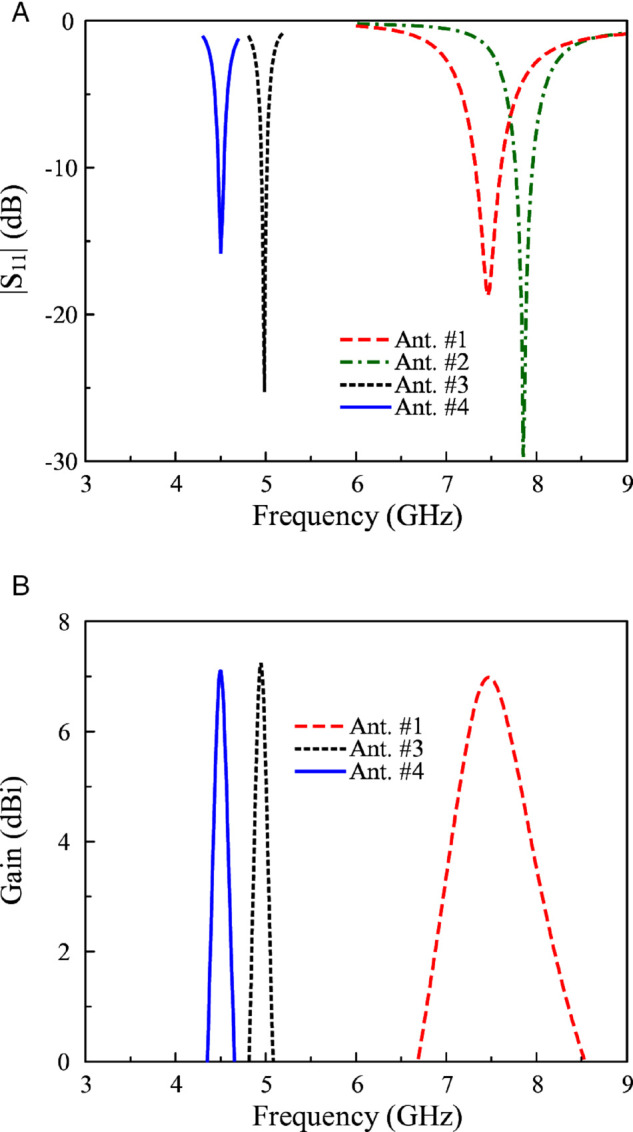
Simulated results of different antennas. (a) |*S*_11_|, and (b) Gain.

The simulated reflection coefficients of Antennas #1, 2, 3, and 4 are presented in Fig 2. For Antenna #1, its resonant length is about half a wavelength at 7.5 GHz. Thus, the simulated |*S*_11_| is lower than –10 dB around this frequency. Antenna #2 is loaded with several gaps and still resonates around 7.5 GHz. For Antenna #3, the operating frequency is moved to a lower frequency range of about 5.0 GHz. The final miniaturized Antenna #4 operates at the lowest frequency of 4.5 GHz. In terms of gain radiation, Fig 2(b) shows the simulated gain radiation of different antenna configurations. In fact, smaller antenna size results in smaller radiating aperture, which leads to lower gain radiation.

## Miniaturization principles

The principle for miniaturization can be explained by the slow-wave theory [32,33]. This demonstrated that by loading more capacitors along the non-radiating edges of the patch antenna, a lower operating frequency can be obtained. Here, Antenna #1 is the conventional patch antenna. Meanwhile, Antennas #2, #3, and #4 become gap-loaded patch antennas when some gaps are inserted to divide the conventional patch into several smaller patches. Noted that for antenna without shorting pins, Antenna #2, the gap is placed along the resonant length of the patch, which acts as an uncoupled gap due to no significant electromagnetic coupling occurring between the two sides of the gap. On the other hand, Antennas #3 and #4, with the presence of the shorting pins, the introduced gaps function as coupled gaps due to the strong electromagnetic field coupling across them. More coupled gaps are obtained for Antennas #4, leading to a lower operating frequency.

**Fig 3:**
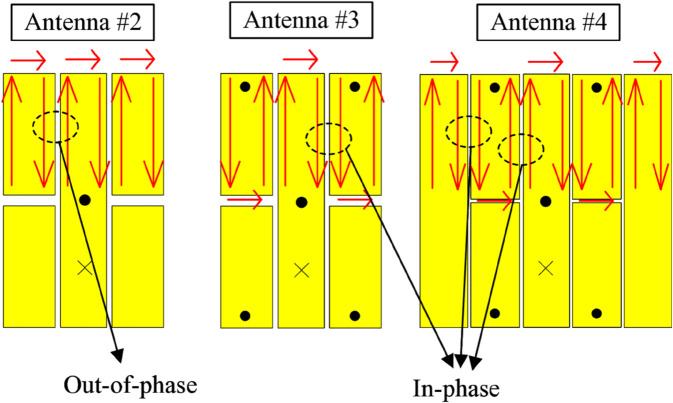
Equivalent magnetic currents flowing on different antennas.

In other words, when the equivalent magnetic currents (*M*) on the gaps are out of phase, their cancellation makes the gaps act as the uncoupled gaps. In contrast, when the magnetic currents are in phase, the coupled gaps are generated. These types of coupled gaps will make the antenna become more capacitive. Antenna #2, without shorting pins, has the out-of-phase *M* around the gaps. Meanwhile, the presence of shorting pins in Antennas #3 and #4 redistributes the *M* on the patch, and the in-phase equivalent magnetic currents are realized around the gaps. The equivalent magnetic currents flowing on Antennas #2, #3, and #4 are presented in Fig 3. According to the transmission line model in [31], the capacitive loading will realize a slow-wave characteristic, which contributes to decreasing the resonant frequency.

**Fig 4:**
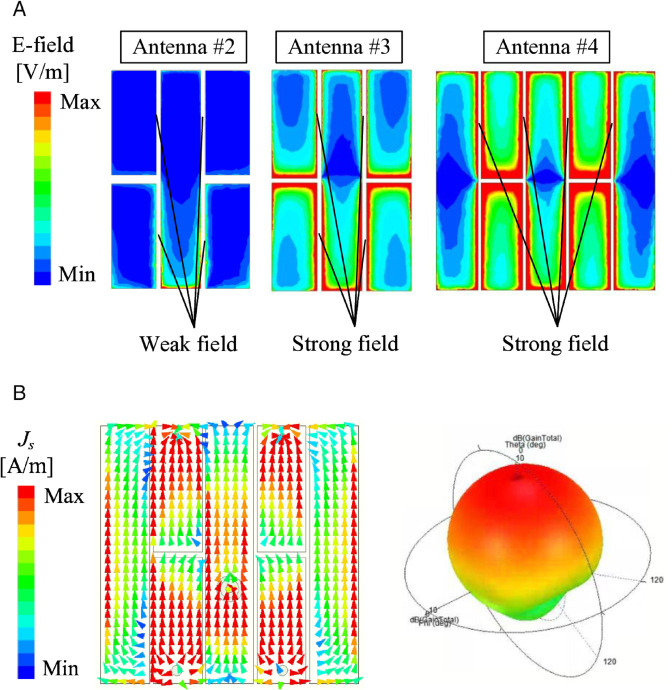
Simulated (a) E-field distribution and (b) vector currents and radiation pattern on different antennas.

For demonstration, the simulated electric field distributions on Antennas #2, #3, and #4 at their resonances are presented in Fig 4. It can be seen obviously that there is a small electromagnetic coupling occurring between the two sides of the gaps in Antennas #2. Meanwhile, Antennas #3 and #4 observe strong coupling fields across the gaps. These loading capacitors make these antennas operate at lower frequencies. Especially, more capacitors are loaded for Antennas #4, which explains why this antenna exhibits the lowest operating frequency. Fig 4(b) also indicates the simulated current distribution at 4.5 GHz and the 3-D radiation pattern at this frequency. As seen, the currents on the patch are in phase, which results in vertically polarized broadside radiation of the fundamental mode, TM_10_.

**Fig 5:**
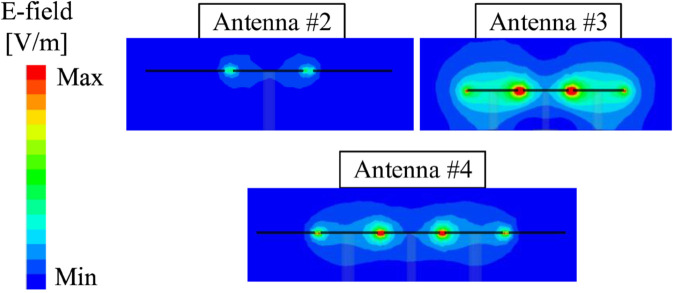
Simulated cross-sectional E-field distribution along the dash line ab.

Fig 5 shows the distribution across the slots of the patch. Here, the cross-sectional E-field distribution along the dashed line ab (shown in Fig 3). It can be seen obviously that Antenna #2 without shorting pins has small E-field distribution across the slots. Meanwhile, Antenna #3 observes significant E-field across the slots. It means that higher capacitance is loaded for Antenna #3, leading to lower resonance. For Antenna #4, additional slots are introduced and accordingly, higher capacitance is obtained for this design. Consequently, Antenna #4 has the lowest operating frequency among the investigated miniaturized patches.

## Antenna operation characteristics

**Fig 6:**
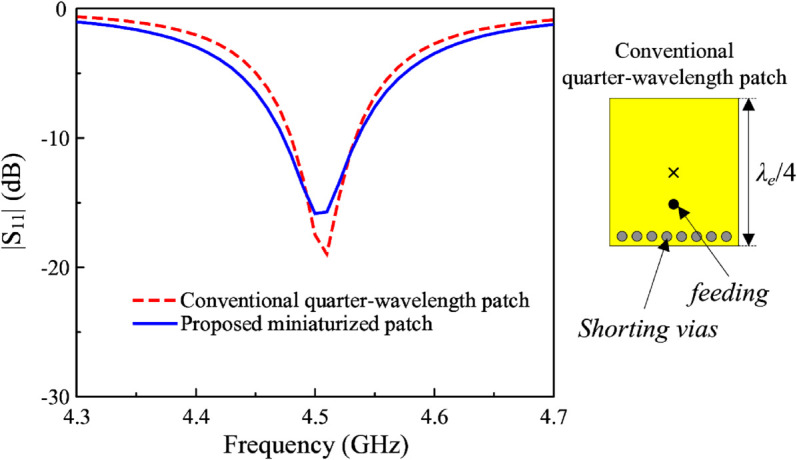
Simulated $\left|S_{11}\right|$ of the conventional and proposed designs.

**Fig 7:**
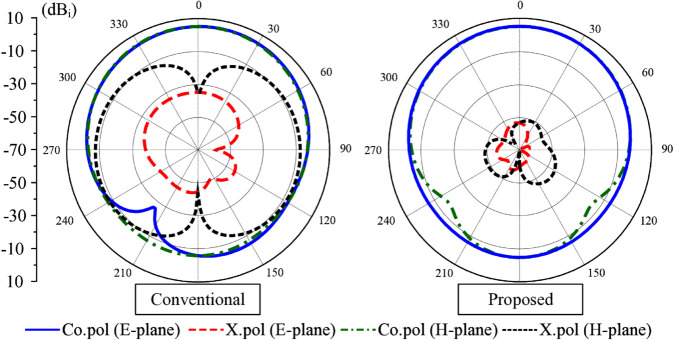
Simulated radiation patterns of the conventional and proposed designs.

The miniaturized patch antenna (Antenna #4) has a resonant length of about a quarter effective wavelength at the desired frequency (4.5 GHz). The performance of this antenna should be compared with that of the conventional quarter-wavelength patch antenna to demonstrate the novel operation characteristic of the proposed miniaturized antenna. Figs 6 and 7 show the performance comparison in terms of reflection coefficient and radiation patterns at 4.5 GHz of both antennas. Noted that in both cases, the ground plane dimensions are fixed at 30 mm × 30 mm. Obviously, both antennas show good matching performance at this frequency. The critical difference comes from the radiation pattern, especially the cross-polarization radiation. The data indicate that the conventional design has large cross-polarization levels. The most significant values in E- and H-planes are –35 and –7.7 dB, respectively. Meanwhile, the proposed miniaturized design has much smaller cross-polarization levels, which are less than –45 dB in both planes. The low cross polarization of the proposed antenna is based on the symmetry of the feeding position. Theoretically, asymmetric feeding disturbs the surface current symmetry and excites unwanted orthogonal modes. Therefore, a carefully optimized and symmetric feed location is essential for achieving low cross-polarization radiation. In the proposed antenna, the main patch is divided into multiple segments. However, the center feeding segment has a narrow width, *w*_1_. The reason is that the input impedance of the rectangular patch increases monotonically from near zero at the patch center to a maximum at the radiating edge, following a cosine-squared law. Smaller width results in higher impedance at the edge, and then the 50-Ω feeding position is closer to the center.

**Fig 8:**
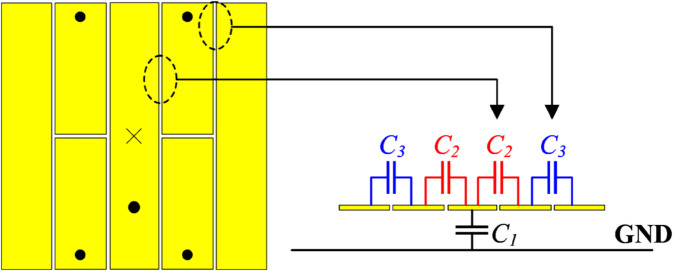
Equivalent circuit of the proposed miniaturized antenna, Antenna #4.

**Fig 9:**
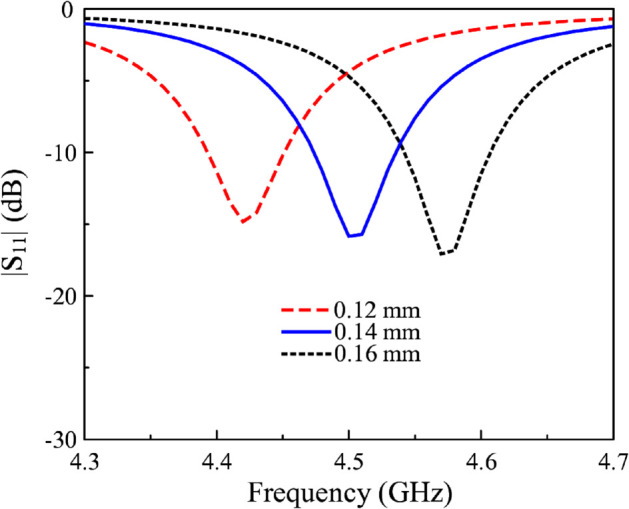
Simulated $\left|S_{11}\right|$ for different widths of the coupled gaps, *g*.

**Fig 10:**
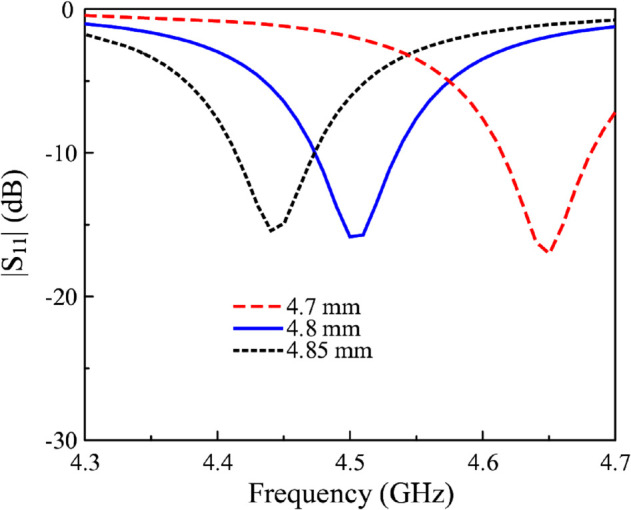
Simulated $\left|S_{11}\right|$ for different lengths of the coupled gaps, *s*.

Fig 8 shows the equivalent circuit in terms of capacitance of Antenna #4. Here, *C*_1_ denotes the capacitance between the directed fed patch and the ground plane. Meanwhile, *C*_2_ and *C*_3_ denote the capacitance of each slot. The total capacitance contained in the proposed antenna strongly depends on the values of *C*_2_ and *C*_3_. Therefore, the gap is the critical parameter to determine the resonant frequency of the proposed antenna. Noted that the equivalent circuit model is employed to qualitatively illustrate the role of the coupled gaps in introducing additional capacitance to the antenna. The impact of this added capacitance on the resonant frequency is evaluated through full-wave electromagnetic simulations. Fig 9 shows the simulated reflection coefficient values against the variation of the gap’s width, *g*. As observed, when *g* increases from 0.12 to 0.16 mm, the resonance tends to move to a higher frequency region. This is because increasing *g* results in lower capacitance, which is inversely proportional to the resonant frequency ($f=1/\sqrt{LC}$). It is noted that for Antenna #3 with the same gap (*g* = 0.14 mm), the resonance is about 4.9 GHz, and this frequency is higher than the operating band of Antenna #4. Finally, the effect of the gap’s length is demonstrated in Fig 10. As observed, this parameter has a substantial impact on the operating frequency of the proposed miniaturized design. A larger value of *s* leads to a larger gap length and a lower capacitance. Accordingly, the resonance shifts upwards to a higher frequency range.

**Fig 11:**
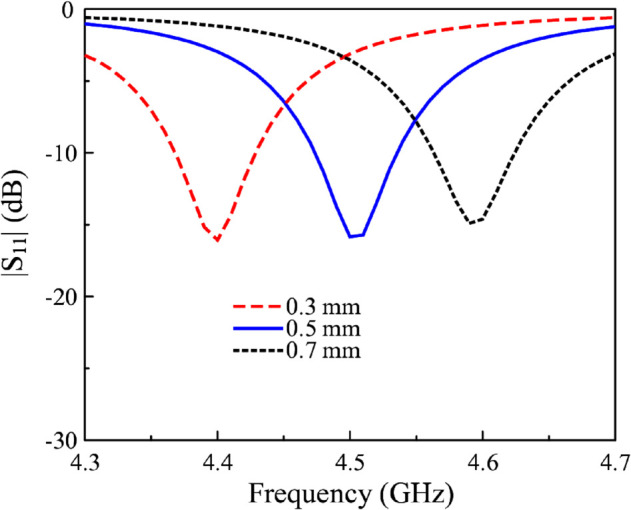
Simulated $\left|S_{11}\right|$ versus changes in the shorting pin’s position, $d_{v}$.

Finally, the effect of the shorting pin’s position is considered. Fig 11 shows the simulated reflection coefficient for different values of $d_{v}$. As seen obviously, when the shorting pins move close to the edge, the antenna resonates in lower frequency region. In contrast, moving the shorting pin far from the edge results in higher operating frequency. The reason behind is that this parameter directly affects the length of the coupled gap, which determine the additional capacitance (*C*_2_ and *C*_3_) to the antenna. According to the parametric studies, the optimization process when applying the proposed method to other frequency band can be divided into two main stages. Firstly, the design should have resonant length of quarter-effective wavelength at the desired frequency. Secondly, tuning the gap dimensions and feeding position to achieve good operating performance.

## Measured results

**Fig 12:**
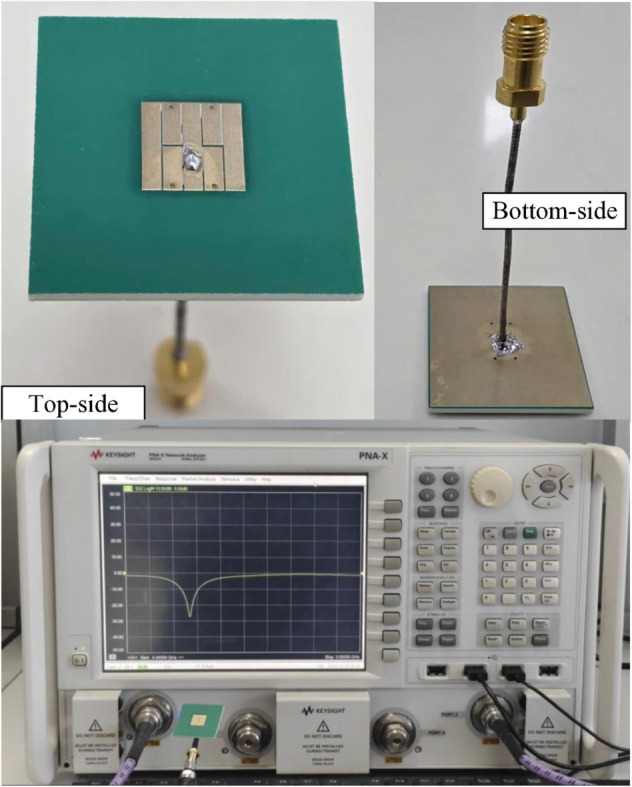
Photos of the fabricated antenna prototype.

The feasibility of the proposed concept is validated by measurement on a fabricated antenna prototype. The antenna is fabricated using printed circuit board (PCB) etching technique. It is noted that the typical fabrication tolerance of standard PCB processes is approximately ±0.02 mm. As shown in Fig 9, the resonant frequency is highly sensitive to variations in the gap dimension. This tolerance can cause the operating resonance shifts from 4.42 to 4.57 GHz, which is still around 4.5 GHz and within the 5G NR *n*79 band (4.4-5.0 GHz). Therefore, the fabrication tolerance of standard PCB processes is acceptable. The photographs in terms of top and bottom views of the fabricated antenna are depicted in Fig 12. The Vector Network Analyzer is employed for measuring the reflection coefficient, |*S*_11_|. Meanwhile, the far-field performance is characterized by the anechoic chamber. Overall, the measurements are pretty identical to those of the simulations. The slight differences could be due to the tolerance in fabrication and misalignment in the measurement setup.

**Fig 13:**
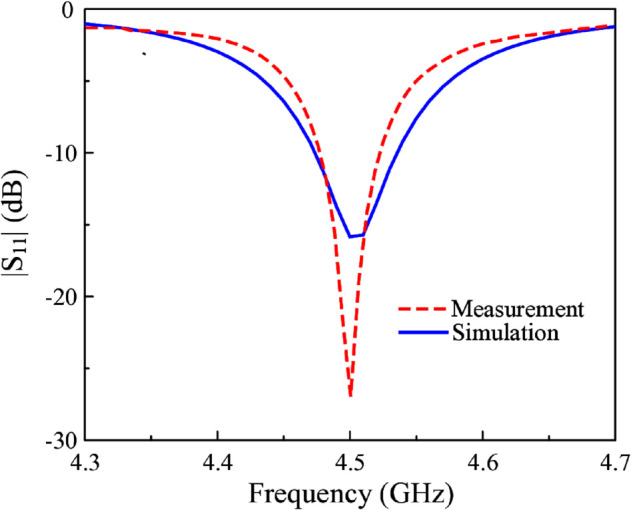
Simulated and measured $\left|S_{11}\right|$ of the proposed miniaturized antenna.

**Fig 14:**
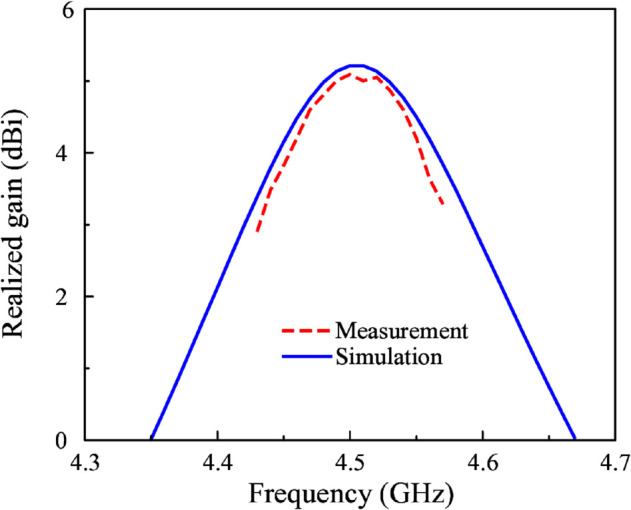
Simulated and measured realized gain of the proposed miniaturized antenna.

The simulated and measured |*S*_11_| results are illustrated in Fig 13. The simulated –10 dB impedance bandwidth ranges from 4.48 to 4.56 GHz. Meanwhile, the measured data is from 4.48 to 4.52 GHz. The proposed antenna exhibits an impedance bandwidth of 40 MHz. In contemporary sub-6 GHz wireless systems around 4.5 GHz, standardized channel bandwidths typically range from 10 to 100 MHz, including 10, 20, 40, 50, 80, and 100 MHz configurations. Therefore, the achieved 40 MHz bandwidth is sufficient to fully support a single 40-MHz channel, demonstrating that the proposed antenna meets the bandwidth requirements of practical sub-6 GHz wireless and IoT applications operating in this frequency range. Fig 14 shows the simulated and measured realized gain values in the broadside direction. As observed within the measured operating bandwidth, the broadside gain lies in the range from 4.2 to 5.1 dBi. The similarity in the simulated and measured gains also confirm the similarity between simulated and measured radiation efficiency. The simulated radiation efficiency of the proposed antenna is higher than 80% across the operating bandwidth.

**Fig 15:**
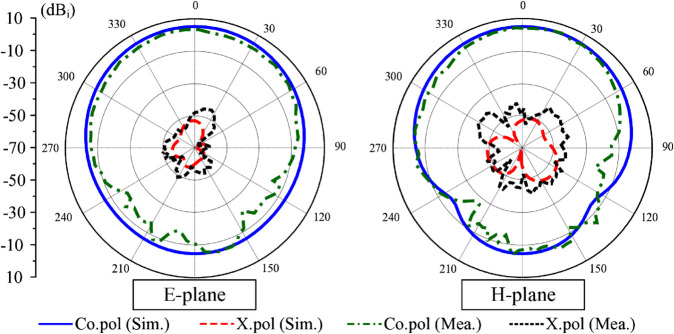
Simulated and measured radiation patterns of the proposed miniaturized antenna.

The simulated and measured radiation patterns at 4.5 GHz are plotted in Fig 15. The results are considered in two principal planes of E- and H-planes (x-z and y-z). It is obvious that the fabricated prototype has symmetrical radiation patterns around the broadside direction. The front-to-back ratio, defined by the difference between the gain in the forward and backward directions, is about 13 dB. Meanwhile, the measured cross-polarization radiations are both lower than –40 dB in both E- and H-planes.

## Performance comparison

Table 2 shows a comparison between the proposed design and other related works to demonstrate the advantages of the proposed miniaturized antenna. The compared data demonstrates obviously that the proposed design shows the smallest cross-polarization radiation and reasonable broadside gain, while its dimensions are one of the most compact designs. The designs in [22,31] have a more compact size than the proposed one, but lower broadside gain and higher cross-polarization level. Meanwhile, several antennas show better broadside gain due to their larger radiating elements. Compared to the similar miniaturization approach of using capacitive load [21–24], the proposed design has strong advantage of low cross-polarization radiation. Besides, the lateral sizes are smaller than [24] and the profile is lower than [21,23]. The design in [22] has the smallest dimensions, but extremely low gain and high cross-radiation level.

**Table 2:** Performance comparison among compact antennas.

Ref.	Overall size (λ)	Radiator size (λ)	Profile (λ)	$\epsilon_{r}$	Freq. (GHz)	Gain (dBi)	X-pol. (dB)
[21]	0.57×0.57	0.15×0.15	0.04	3.4	8.5	5.4	≤−25
[22]	0.35×0.24	0.09×0.09	0.02	2.2	2.4	2.7	≤−15
[23]	0.48×0.48	0.16×0.16	0.04	3.4	9.0	5.2	≤−15
[24]	0.53×0.47	0.23×0.23	0.02	2.6	3.5	5.0	≤−22
[25]	0.45×0.45	0.14×0.14	0.02	4.2	2.45	≤2	≤−20
[26]	N/A	0.20×0.20	0.01	2.2	3.2	3.2	≤−20
[30]	0.82×0.82	0.22×0.14	0.02	2.2	1.65	6.2	≤−21
[31]	0.80×0.80	0.10×0.09	0.02	3.6	2.85	2.6	≤−26
**Prop.**	0.45×0.45	0.15×0.15	**0.02**	**3.5**	**4.5**	**5.1**	≤−40

## Antenna with further miniaturization

**Fig 16:**
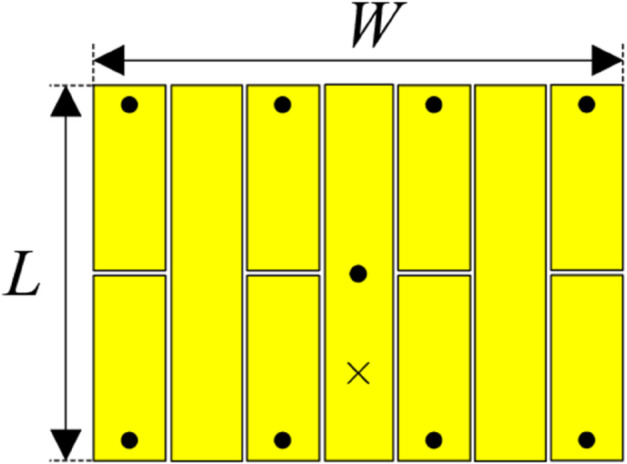
Geometry of the further miniaturized patch antenna.

**Fig 17:**
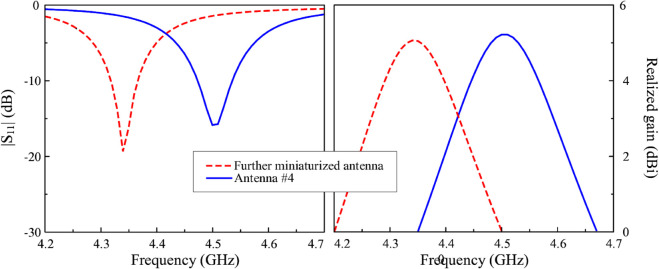
Simulated $\left|S_{11}\right|$ and broadside gain of different miniaturized antennas.

**Fig 18:**
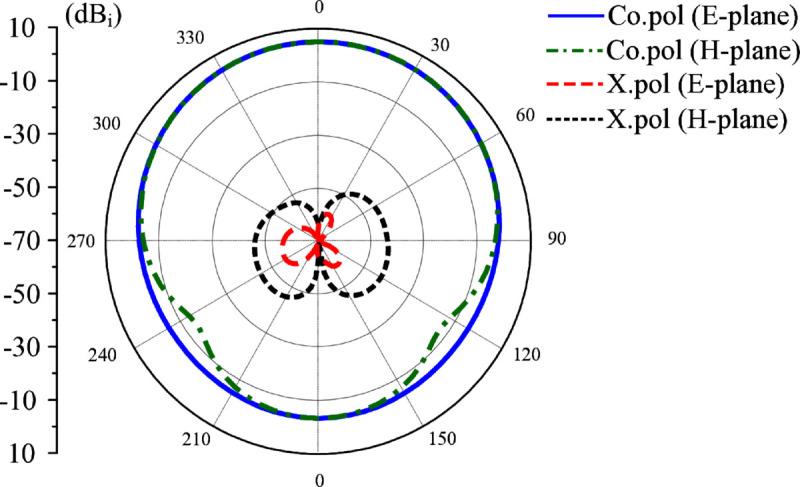
Simulated radiation patterns at 4.34 GHz of the further miniaturized patch antenna.

Further size reduction can be obtained when more coupled gaps are inserted in the antenna. Fig 16 shows the geometry of the antenna with more coupled gaps. The overall dimensions of the radiating element are 9.8 mm × 13.1 mm. The simulated |*S*_11_| and broadside gain of the further miniaturized antenna are shown in Fig 17. It can be seen that the design with more coupled gaps exhibits a lower operating frequency than Antenna #4, 4.34 GHz compared to 4.5 GHz. Meanwhile, the gain radiation patterns plotted in Fig 18 also demonstrate that the more coupled gap-loaded antenna still has a good broadside beam and extremely low cross-polarization radiation. Compared to Antenna #4 with a similar ground plane size of 30 mm × 30 mm, Antenna #4 performs higher broadside gain and lower back radiation.

## Conclusion

This work introduces a miniaturization method for a microstrip patch antenna operating in the fundamental mode. The patch is modified by inserting multiple gaps to divide the patch into multiple smaller patches. Then, the shorting via is utilized to produce the coupled gap-loaded antenna. These coupled gaps function as the capacitor, which makes the resonance shift notably toward a lower frequency. The optimized radiator, having a physical size of about a quarter of the effective wavelength at 4.5 GHz, achieves good impedance matching around 4.5 GHz and delivers a broadside gain of roughly 5.0 dBi. Furthermore, the design demonstrates exceptionally low cross-polarization radiation, which is below –45 dB.
